# Discovery of microRNAs during early spermatogenesis in chicken

**DOI:** 10.1371/journal.pone.0177098

**Published:** 2017-05-22

**Authors:** Lu Xu, Qixin Guo, Guobin Chang, Lingling Qiu, Xiangping Liu, Yulin Bi, Yu Zhang, Hongzhi Wang, Wei Lu, Lichen Ren, Ying Chen, Yang Zhang, Qi Xu, Guohong Chen

**Affiliations:** 1 College of Animal Science and Technology, Yangzhou University, Yangzhou, Jiangsu, China; 2 Poultry Institute, Chinese Academy of Agricultural Sciences, Yangzhou, Jiangsu, China; Qingdao Agricultural University, CHINA

## Abstract

Spermatogenesis is a complex process that involves many elements. However, until now, little is known at the molecular level about spermatogenesis in poultry. Here we investigated microRNAs and their target genes that may be involved in germ cell development and spermatogonial in chicken. We used next-generation sequencing to analyze miRNA expression profiles in three types of germline cells: primordial germ cells (PGCs), spermatogonial stem cells (SSCs), and spermatogonia (Sp) during early stage of spermatogenesis. After validated the candidate miRNAs and corresponding genes’ expression in three types of cells, we found 15 miRNAs that were enriched 21 target genes that may be involved in spermatogenesis. Among the enriched miRNAs, miR-202-5p/3p were up-regulated in the Sp library and down-regulated in the PGCs library. Through RT-qPCR and Dual-Luciferase reporter assay, we confirmed that miR-202-5p bind to *LIMK2* and involved in germ cell development. Collectively, we firstly discover the novel miRNAs, like miR-202-5p, and its related genes and pathways, expressed during the early spermatogonial stage in chicken, which will provide new clues for deciphering the molecular mechanism of the miRNAs regulating germline stem cell differentiation and spermatogenesis in chicken.

## Introduction

Azoospermia has been reported to cause amount of losses in the chicken breeding industry because of the large number of chickens’ weed out. However, so far, the mechanisms involved in the spermatogenesis process in chicken are not well understood. To form functional sperm, immature male germ cells undergo a coordinated set of events that are collectively called spermatogenesis. Many types of cells, including Sertoli cells, peritubular myoid cells, and Leydig cells are involved in this process [1]. Primordial germ cells (PGCs), spermatogonial stem cells (SSCs), and spermatogonia (Sp) belong to early stage of spermatogenesis [1]. PGCs are the primary germ cells that are established during development and are immediate precursors of both the oocytes and Sp cells [2]. Some of PGCs undergo several processes to become gametogenesis-competent cells, which have the capacity for meiotic initiation and sexual differentiation [3]. SSCs are present in the testicles and are already present at birth [4,5]. They are the progenitors of male gametes and critical for the process of spermatogenesis [6]. Unlike the former two germ stem cells, Sp are somatic cells and early developmental stages cells that subsequently differentiate into spermatocytes. It has been reported that a clot composed of fowl plasma and embryo extract permitted some mitotically active Sp cells in cultured newborn mouse testes to progress to the pachytene stage [7].

Recently, many studies have suggested that microRNAs (miRNAs) play major roles in germ line stem cells and spermatogenesis. In bovine, large numbers of miRNAs were detected in spermatozoa and their differential expression in sperm from high versus low fertility bulls suggested that these miRNAs play important roles in regulating the mechanisms of bovine spermatozoa [8]. In human, it has been reported that miR-122 may influence spermatozoa-like cells by suppressing *TNP2* expression and inhibiting the expression of proteins associated with sperm development [9]. miRNAs have also been found to affect germline stem cell development; for example, miR-181a* was reported to play two different functional roles in chicken PGCs by binding to two different transcripts and prevented PGCs entering meiosis [10]. In mouse, miR-21 was shown to be important in maintaining the SSC population; suppression of miR-21 resulted in large numbers of germ cells undergoing apoptosis and significantly reduced the number of donor-derived colonies of spermatogenesis [11]. Gonads are complex tissues made up of different types of cells and this can make sequencing results difficult to interpret. In addition, there was little reports have suggested that spermatogenesis from epigenetic in poultry. In this study, we used three types of cells (PGCs, SSCs, and Sp) from chicken to examine the relationship between miRNAs and spermatogenesis. We analyzed the miRNA profiles of these cells during the early spermatogenesis stage through small RNA-sequencing, and investigated several key miRNAs and their target genes that were associated with germ cell development and spermatogenesis. We also propose an explanation for this process from an epigenetic viewpoint.

## Material and methods

### Ethics statement

All the animal experimental procedures were approved and guided by the Academic Committee of Yangzhou University according to the Management Measures of Laboratory Animal in Jiangsu Province (Permit Number: 45, Government of Jiangsu Province, China) and the US National Institute of Health Guidelines (NIH Pub. No. 85–23, revised 1996).

### Samples

2000 freshly fertilized eggs and twenty-five 28-week-old sexual mature male Langshan chickens were purchased from the Poultry Institute, Chinese Academy of Agricultural Sciences (Yangzhou, China). 1000 eggs, hatched for 5.5 days, were used to isolate PGCs from the gonads of embryos and remaining eggs, hatched for 18 days, were used to isolate SSCs from the testis of embryos. Sp cells were isolated from sexual mature testicle of the male chickens and sorted by flow cytometry, as described previously [12,13].

According to the rules of animal welfare, twenty-five 28-week-old sexual mature male Langshan chickens were stay in 25°C (average temperature) environment. Each chicken feed in one cage and cage volume was 35*38*42 centimeters. They feed with complete diet three times a day. The treatment of twenty-five chickens were as followed: firstly, we use Xylazine Hydrochloride Injection to anesthetize the chicken with the volume of medical 0.4ml/kg. 2 minutes later, the wings and tail feathers fall down. 8 minutes later, the eyes were closed and fall into the sleep. Secondly, remove the right abdomen feathers with surgical scissors and clean the skin with 75% alcohol. Then gash skin tissues by scalpel to and find the testis. Finally, we performed wound closure to chicken. It would take 35 minutes to finish this surgery and the chicken kept sleeping during surgery. Thirdly we used brachial vein injection to treat with chicken euthanasia. Find brachial vein under the wing and clean the skin with alcohol to make the vein expand, then we inject air into the vein with injector. According to Animal Management Rules of Yangzhou University, we transfer these chickens to that office. All the treatment were performed in aseptic environment and we covered with protection suit.

#### Preparation of PGCs and SSCs

We isolated two cell types described by Li *et al*.[14] and Sun *et al*. [15]. Both cell types were cultured in standard stem cell knock-out DMEM culture medium (Life Technologies, Shanghai, China) supplemented with 7.5% fetal calf serum (FBS) (Gibco, US origin, USA), 2.5% chicken serum (Life Technologies, New Zealand), 2 mmol/L L-glutamine (Gibco, Life Technologies, China), 1 mmol/L sodium pyruvate (Gibco, Life Technologies, China), 5.5 × 10^−5^ mol/L β-mercaptoethanol (BBI, Toronto, Ontario, Canada), 0.1 mmol/L non-essential amino acids (Gibco, Life Technologies, Shanghai, China), 5 ng/mL human stem cell growth factor (Sigma-Aldrich, China), 10 ng/mL mouse leukemia inhibitory factor (Sigma-Aldrich, China), 10 ng/mL fibroblast growth factor (Sigma-Aldrich, China), and 100 U/mL of a penicillin-streptomycin combination (Life Technologies, China). Cells were cultured in a CO_2_ incubator (Thermo Fisher Scientific, China) at 37°C and 5% CO_2_. PGCs colonies were identified using mouse anti-chicken C-kit antibody stain (Santa Cruz Biotechnology, Santa Cruz, CA, USA; 1:50) with goat-anti-mouse IgM-FITC (anti-fluorescein isothiocyanate) as the second antibody (Santa Cruz Biotechnology; 1:50) according to the manufacturer’s instruction. The SSCs were identified using rabbit polyclonal antibody to integrin alpha 6 conjugated to FITC (Biorbyt Biotechnology, Biorbyt, UK; 1:50) stain according to the manufacturer’s instructions. PGCs and SSCs were screened with the c-kit antibody and integrin alpha 6 antibody, respectively, using a FACSAria flow cytometer (BD Biosciences, San Jose, CA, USA).

#### Preparation of spermatogonium

Spermatogonium were isolated from sexual mature chicken’s testicle. According to Chang et al. and Xu et al. found that Sp single-cell suspensions from testicular tissues were prepared well and isolated successfully by flow cytometry we obtain the single-cell suspension from testicle [12,13]. The Sp cells were dyed with 2-(4-amidinophenyl)-6-indolecarbamidine dihydrochloride (DAPI) (Beyotime Biotechnology, Shanghai, China) during the flow cytometer process.

### Three types of libraries construction and sequencing

Total RNA was isolated from three types of cells (PGCs, SSCs, and Sp) using a Total RNA Isolation Kit (Tiangen, Shanghai, China) according to manufacturer’s instruction. Each test sample used 3 μg total RNA for small RNA-sequencing. Agilent 2100 Bioanalyzer (Agilent Technologies, Santa Clara, CA, USA) was used for evaluated the RNA samples. 5' and 3' adaptors were ligated to small RNA fractions. After amplification, the quantity and quality of the sequences (140–150 bp) in the PGC, SSC, and Sp cell libraries were measured using a Qubit^®^ 2.0 Fluorometer and Agilent 2100 Bioanalyzer (Agilent Technologies). Small RNA-sequencing was performed on a HiSeq 2500 platform at the Shanghai Biotechnology Co., Ltd (Illumina, Shanghai, China).

### Bioinformatics analysis

The raw data were filtered by removing adaptor-ligated contaminants, low-quality reads (Q-value <20), and short read tags (<18 bp) using the FASTX-toolkit (version 0.0.13.2) in FastQC (http://www.bioinformatics.babraham.ac.uk/projects/fastqc/) to obtain high-quality reads. Briefly, to remove the non-target small RNAs, the reads were aligned with sequences in the miRbase, ncRNA, and Rfam databases, respectively, using the CLC Genomics Workbench. First, we identified the miRNAs that occurred frequently in the three libraries [16]. Then, to find the target genes, we mapped the miRNA sequences onto the reference chicken genome from the National Center for Biotechnology Information (NCBI; Gallus gallus v.4) using miRDB software with the default parameters, except the score was set to >= 140, and the free energy was set to <= −20 kcal/mol. MiRNA expression levels were calculated as transcripts per million reads. Gene Ontology (GO) and Kyoto Encyclopedia of Genes and Genomes (KEGG) pathways were assigned to the miRNA target genes. A GO functional analysis of the target genes was conducted using DAVID (https://david.ncifcrf.gov/) and WEGO (http://wego.genomics.org.cn/cgi-bin/wego/index.pl). The KEGG pathways were analyzed using SBC Analysis System (SAS, Shanghai Biotechnology Co., Ltd, Shanghai, China).

Furthermore, we compared the miRNA expression levels in the three libraries as follows: SSC vs. PGC, Sp vs. SSC, and Sp vs. PGC. Differently expressed miRNA in the three groups comparisons were identified based on |Log (Fold Change)| ≥1 [17].

### Quantitative RT-PCR

Both candidate genes (CWBIO, Beijing, China) and microRNAs (Takara) quantitative RT-PCR (RT-qPCR) using SYBR green was performed according to the manual. Chicken GAPDH and U6 were used as a control. Reverse transcriptase reactions included 500 ng of total RNA per sample. Quantitative PCR reactions were used to calculate the relative fold-change in accordance with the ΔΔCT method. Where appropriate, comparisons of gene expression levels were analyzed by ANOVA using SPSS19.0 and visualized with SigmaPlot 12.5. All miRNAs primers were designed by TIANGEN Biotech CO. LTD.

### Cell transfection

According to manual of Lipofectamine 2000 (Invitrogen, Shanghai, China), we transfected the miR-202-5p mimics and inhibitor in PGCs and DF-1. After 48 hours, collected the cells and detected relative expression of miR-202-5p and related genes. Each group had 8 biological replicates.

### Dual-Luciferase reporter assay

We construct the luciferase vectors to verify the relationship between miR-202-5p and LIMK2. The primer of pGL3-LIMK2: F- GCCACCGCTAGCTCTCTGGCCTTTTCAGGCTTC; R- GCCACCAAGCTTCCACCCTGTCACCTCGTTTC. Co-transfected the pGL3-LIMK2, pRL-TK and miR-202-5p mimics via Lipofectamine 2000 in DF-1. Ratio of 50:1 for pGL3-LIMK2: pRL-TK and the 60nM for miR-202-5p mimics, pGL3-Basic as control. In addition, we construct the pGL3-LIMK2 mutant type which mutate the combination site and this vector was synthesis via Shanghai Generay Biotech Co., Ltd. According to manual of Dual-Luciferase Reporter Assay System (Promege, America), we test whether miR-202-5p bind in *LIMK2* or not. Each group had 4 biological replicates.

## Result

### Identification of PGCs, SSCs, and Sp cells

**Fig 1:**
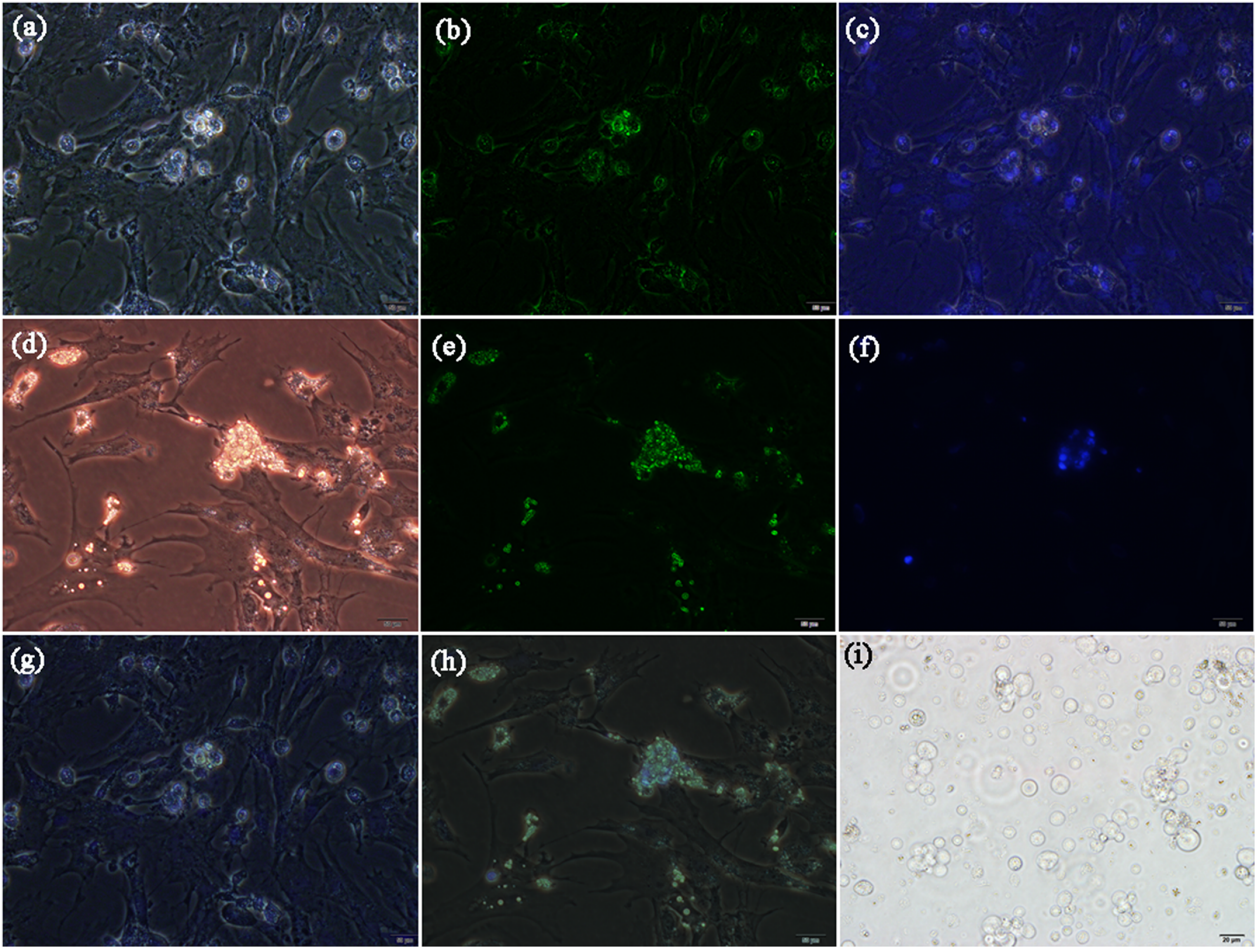
Identification of PGCs, SSCs, and Sp cells by flow cytometry sorting. The primordial germ cells (PGCs) and spermatogonial stem cells (SSCs) were cultured for 48 hours. The spermatogonia (Sp) cells were isolated directly from testes. (**a**) The PGCs capture in light (200×), (b) the PGCs identified with c-kit antibody marked with FITC and capture in dark (200×), (c) the PGCs stain with DAPI and capture in dark (200×), (g) it was a merge figure include (a-c). (d) The SSCs capture in light (200×), (e) the SSCs marked with integrin alpha 6 antibody (FITC) (200×), (f) the SSCs stain with DAPI and capture in dark (200×), (h) it was a merge figure include (d-f). (**c**) The Sp cells were collected by flow cytometry (200×). (i) The Sp cells were isolated and recorded in light. Bar = 20 μm.

We performed the immunofluorescence on PGCs (mouse anti-chicken C-kit antibody) and SSCs (rabbit polyclonal antibody to integrin alpha 6) (Fig 1). Both PGCs and SSCs were cultured for 48 hours. Sp were larger than PGCs and SSCs (Fig 1).

### Sequencing and identification of miRNAs

**Fig 2:**
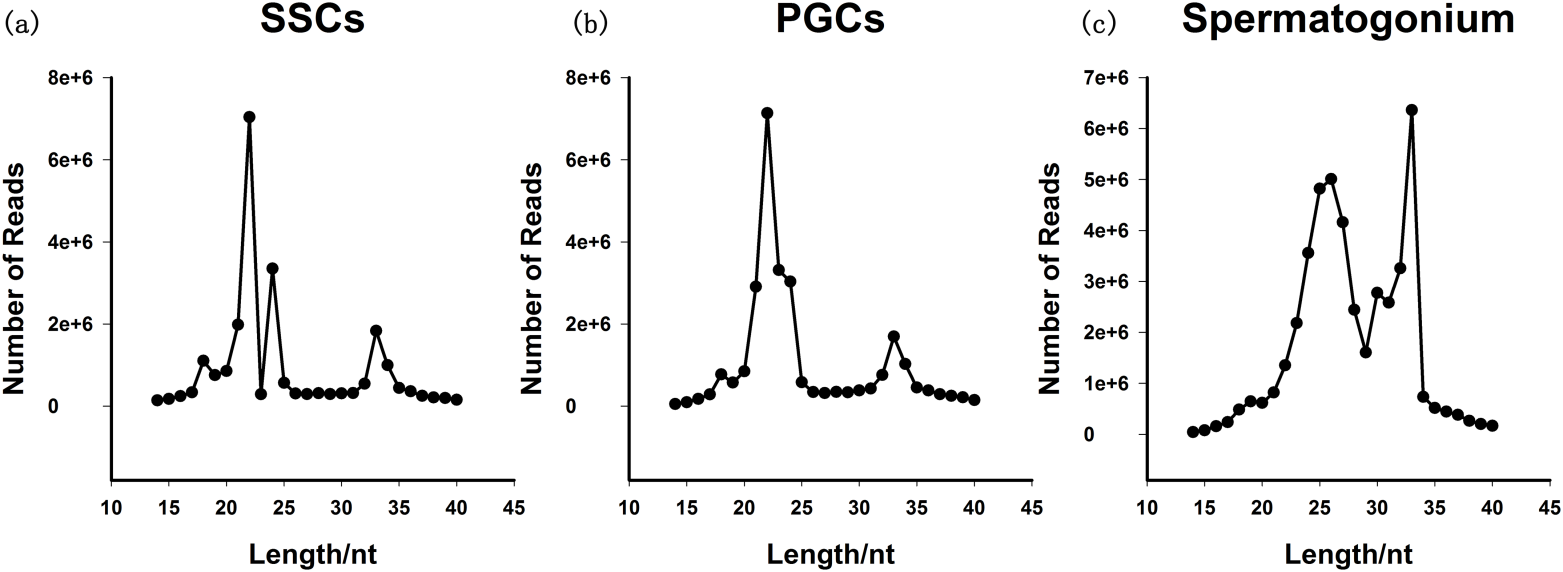
Length distributions of the small RNAs in the PGCs, SSCs, and Sp libraries. (a) The SSCs library, (b) the PGCs library, (c) the spermatogonium library.

**Fig 3:**
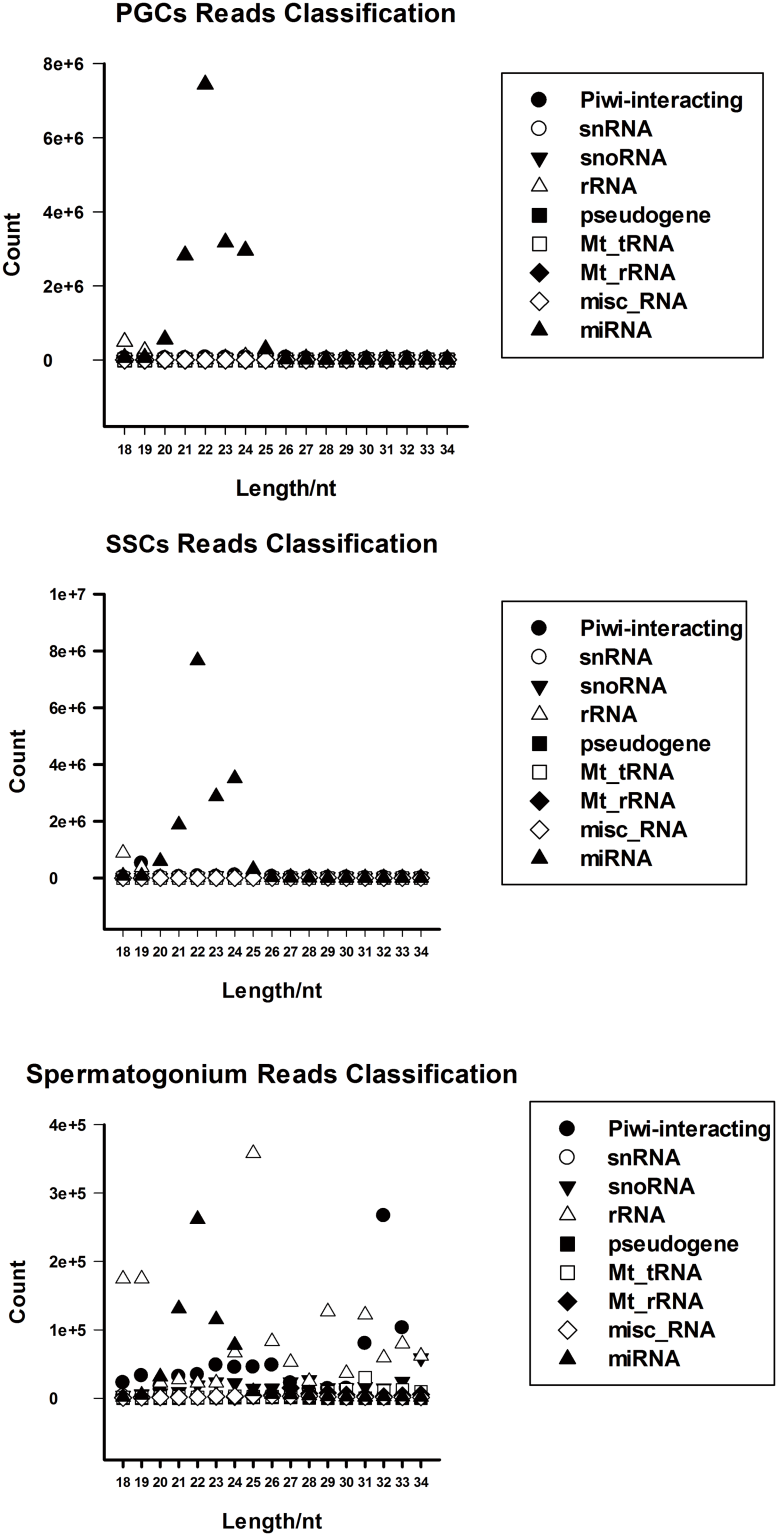
Classification and annotation of the small RNAs in the PGCs, SSCs, and Sp libraries. (a) The PGC library, (b) the SSC library, and (c) the spermatogonium library. Different colors indicate the different small RNA groups.

Large amount of reads were obtained from sequencing and all the effective ratio was > 95% (Table 1). After length distribution analysis, two peaks present in Fig 2 and we mainly focus on first peak [18,19]. MiRNAs have been reported previously to range mainly from 21–24 nt in PGCs and SSCs, and from 24–27 nt in Sp cells (Fig 2). According to database searches, nine groups were classified and annotated that miRNAs, misc_RNAs, Mt_rRNAs, Mt_tRNAs, pseudogenes, rRNAs, snoRNAs, snRNAs, and Piwi-interacting RNAs (Fig 3). The distribution of the different types of RNAs was different in the different cell types.

**Table 1:** An overview of sequencing.

Library	Clean Reads	Mapped Reads	Uniq Reads	Known miRNAs	Novel miRNAs	Effective Ratio
PGCs	30,851,348	19850783	1864758	12,521,222	5251	98.22%
SSCs	31,461,002	20074827	1654396	13,039,779	5304	98.03%
Sp	46,273,957	19022593	8650846	514,795	21015	98.14%

**Fig 4:**
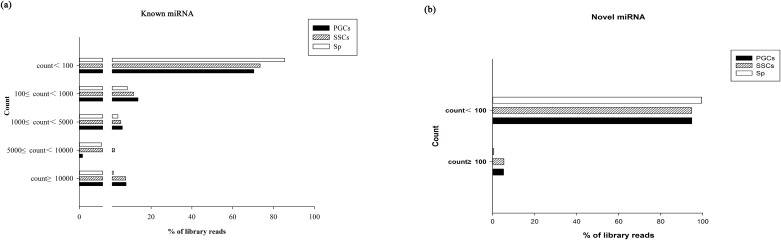
Abundance distributions of known and novel miRNAs in the PGCs, SSCs, and Sp libraries. (a) The known miRNAs and (b) the novel miRNAs. We chose the most abundant in three libraries to analyze.

A total of 2547 different known miRNAs were identified in the three type of cells; 874 were in the PGCs, 884 were in the SSCs, and 789 were in the Sp cells. We divided the known miRNAs into five groups based on their counts in the three libraries as follows: >= 10000, < 10000 and >= 5000, < 5000 and >= 1000, < 1000 and >= 100, and < 100 (Fig 4a). The most frequently sequenced known miRNA in the PGCs and SSCs libraries was miR-21-5p, which made up 25.87% and 28.23% of the reads, respectively, while in the Sp library miR-100-5p was the most frequently sequenced, making up 11.93% of the reads. The miRNAs with counts ≥10000 were listed in S1 Table. In addition to the known miRNAs, 1228 novel miRNAs were identified in the three types of cells; 172 were in the PGCs, 130 were in the SSCs, and 926 were in the Sp cells. Twenty-one of the novel miRNAs had counts >100 in the three types of cells; nine were in the PGCs, seven were in the SSCs, and five were in the Sp cells (Fig 4b).

### Analysis of the most abundant mature miRNAs

**Fig 5:**
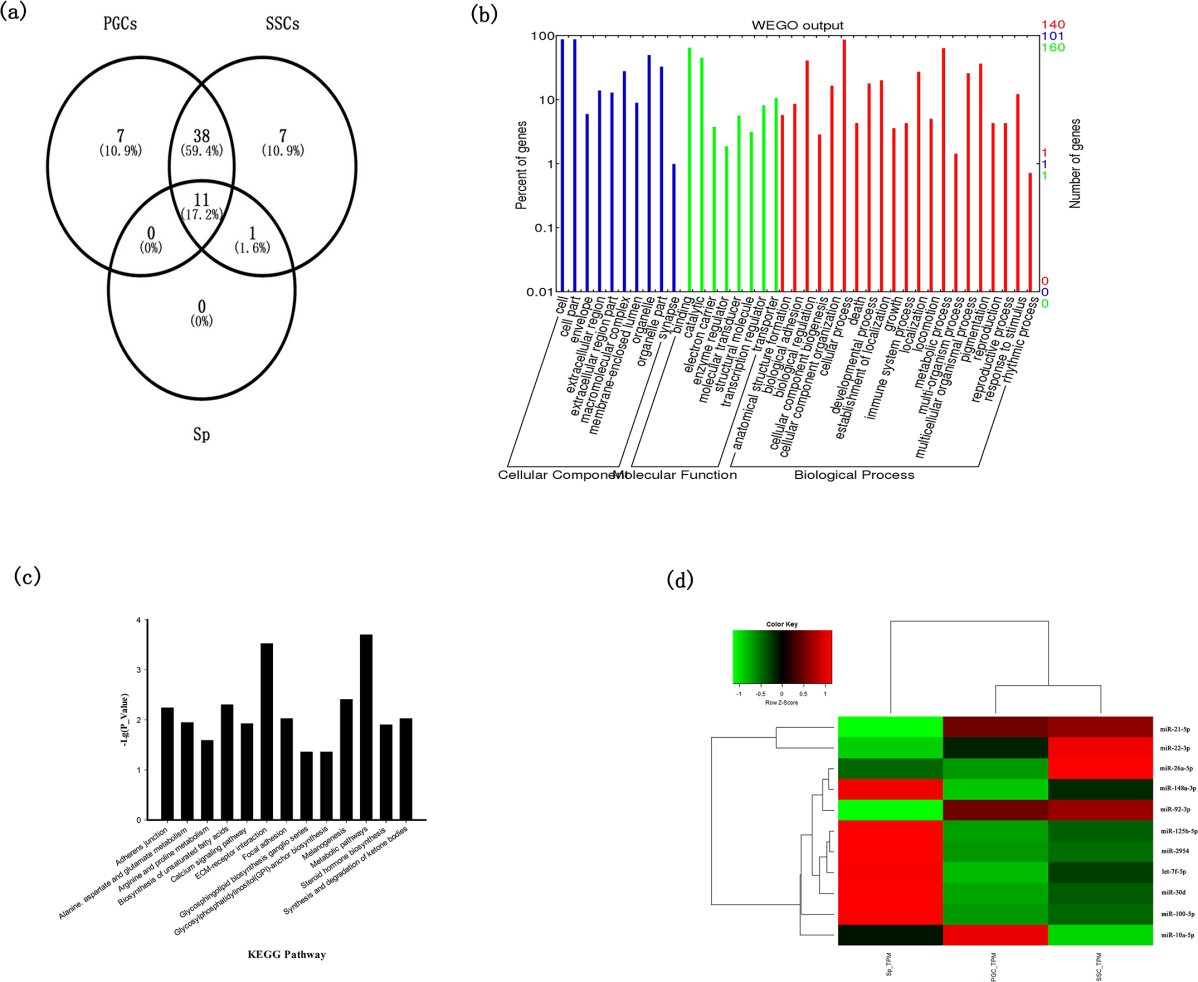
Analyses of the most abundant mature miRNAs in the PGCs, SSCs, and Sp libraries. (a) Venn analysis of the group with counts ≥10000. (b) GO analysis of the target genes of 11 common miRNAs. (c) KEGG pathway analysis of the target genes of 11 common miRNAs. (d) Expression patterns of 11 common miRNAs in the three types of cells. Red indicates up-regulated; green indicates down-regulated.

Venn analysis of the group with counts >= 10000 (Fig 5a) showed that 11 of the miRNAs in this group were present in the three libraries, including miR-21-5p and miR-100-5p (marked with red in S1 Table). We identified 333 target genes for the 11 miRNAs using miRDB. The GO functional analysis of the target genes was shown in Fig 5b. Among the 333 genes, 121 genes were annotated with terms in the cellular process category; 6 of 121 genes were associated with reproduction, including sexual reproduction and reproductive process, and 25 of 121 genes were involved in development process, including reproductive development, embryonic development, and stem cell differentiation. The KEGG pathway analysis revealed 88 pathways that were enriched in the 333 target genes and 13 pathways showed difference (*P* <= 0.05) (Fig 5c). A complete list of the KEGG pathways is given in S2 Table. Among of these pathways 13 pathways, we mainly focus on Melanogenesis, Steroid hormone biosynthesis which related with germ cell development or spermatogenesis. According to WEGO results, we identified 13 genes that are known to play key roles in germ cell maintenance or proliferation and spermiogenesis, namely *PTPRU*, *LAMA2*, *ITGB2*, *RELN*, *DICER1*, *ITGB1*, *BRCA2*, *NR2C2*, *ACOX1*, *TBX2*, *CDH4*, *CTGF*, and *HIF1A*. The miRNAs predicted to target these genes were mainly miR-21-5p, miR-100-5p, miR-148a-3p, let-7f-5p, and miR-10a-5p. The expression patterns of the 11 miRNAs in the three types of cells are shown in Fig 5d.

In Sp there were 12 miRNAs that count >= 10000 but 11 miRNAs appeared in PGCs, SSCs and Sp. The left one was miR-202-5p which present in SSCs either. Thus we analysis miR-202-5p. There were 18 genes targeted by miR-202-5p. *LIMK2* was one of the 18 genes and we would like analysis it in next step.

### Analysis of the differentially expressed miRNAs and their target genes

**Fig 6:**
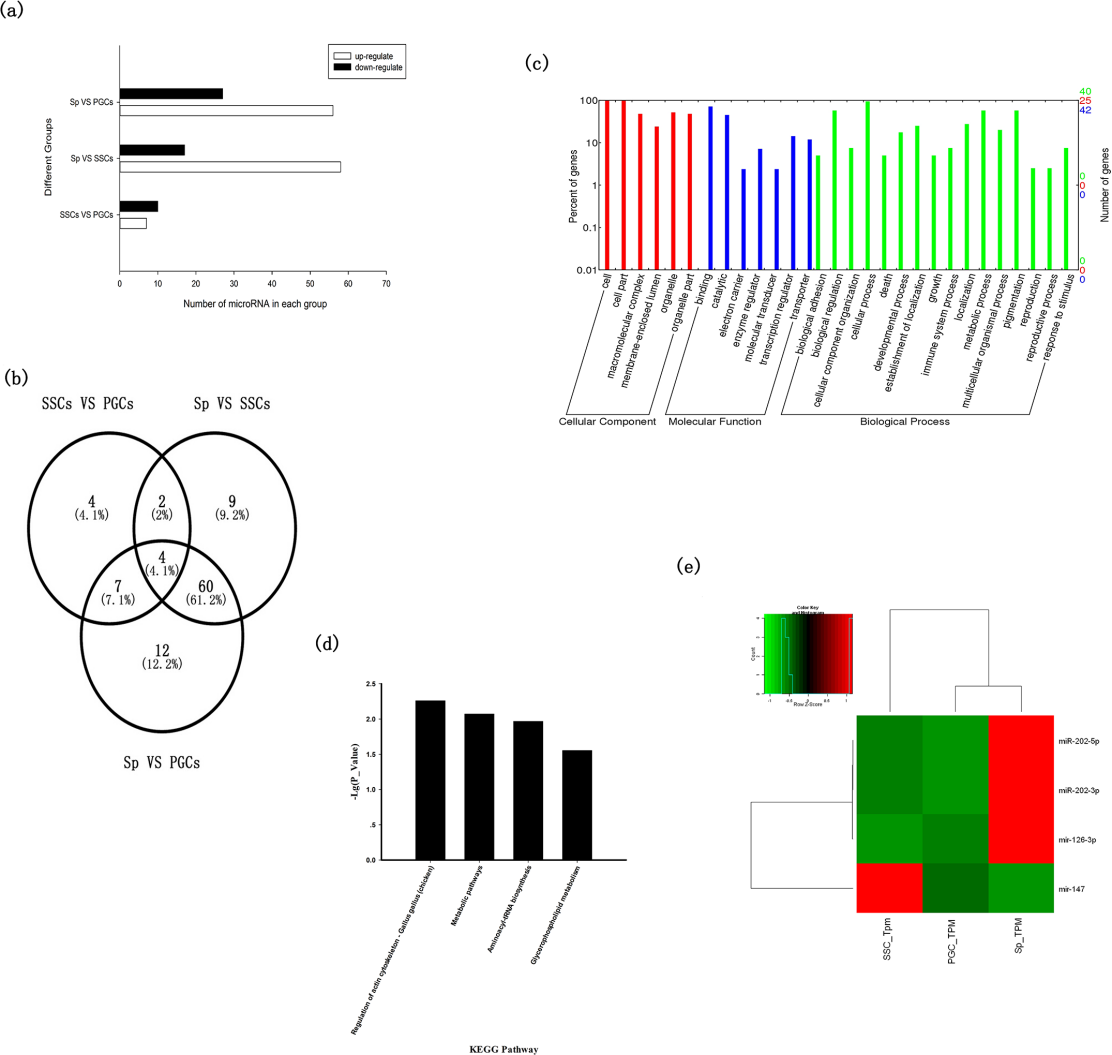
Differentially expressed miRNAs and their target genes. (a) Numbers of miRNAs in each comparison. (b) Venn analysis of the differentially expressed miRNAs in three comparisons. (c) GO analysis of the target genes of four common differentially expressed miRNAs (miR-202-3p, miR-202-5p, miR-147, and miR-126-3p). (d) KEGG analysis of the target genes of four common differentially expressed miRNAs (miR-202-3p, miR-202-5p, miR-147, and miR-126-3p). (e) Expression patterns of four common differentially expressed miRNAs (miR-202-3p, miR-202-5p, miR-147, and miR-126-3p). Red indicates up-regulated; green indicates down-regulated.

We detected 175 differentially expressed miRNAs in the three comparisons (SSCs vs. PGCs, Sp vs. SSCs, and Sp vs. PGCs), as shown in Fig 6a. Venn analysis of these miRNA identified 4 miRNAs expressed in all samples analyzed: miR-202-3p, miR-202-5p, miR-147 and miR-126-3p (Fig 6b). MiR-202-5p/3p was up-regulated in SSCs vs. PGCs, Sp vs. SSCs, and Sp vs. PGCs. And miR-147 was up-regulated in SSC vs. PGC and down-regulated in the other two comparisons. Conversely, miR-126-3p was down-regulated in SSC vs. PGC and up-regulated in the other two comparisons. MirDB predicted 78 target genes for the four common differentially expressed miRNAs. Among them miR-202-3p was predicted to target 34 genes, miR-202-5p targeted 18 genes, miR-147 targeted 17 genes, and miR-126-3p targeted 9 genes. GO analysis of the 78 target genes identified 38 genes involved in cellular process, 7 involved in development, including embryo development, 2 associated with growth, and only one involved in reproduction, including sexual reproduction, gamete generation, and spermatogenesis (Fig 6c). KEGG analysis of the target genes of the 4 common differentially expressed miRNAs identified 36 pathways enriched in these genes and 4 pathways showed the difference (*P* < 0.05) (Fig 6d), that Regulation of actin cytoskeleton, Metabolic pathways, Aminoacyl-tRNA biosynthesis and Glycerophospholipid metabolism. A complete list of the KEGG pathways given in S3 Table.

In accordance with above analysis, we identified 2 pathways and several genes *DVL3*, *LIMK1*, *LIMK2*, *ANAPC10*, *IKZF1*, *HOXD4*, *NTRK2*, and *PTGES2* associated with germline stem cell proliferation or spermatogenesis. The GO analysis suggested that these genes played different roles in different processes. *DVL3* was involved in intracellular signaling cascade; *LIMK1* and *LIMK2* were associated with cell growth and sexual reproduction, respectively, as well as regulation of actin cytoskeleton; *ANAPC10* was involved in cell cycle; *IKZF1* was involved in sperm cell differentiation; *HOXD4* was associated with embryonic organ development; *NTRK2* was annotated as binding the growth factor, and *PTGES2* was involved in regulation of biological quality which included the homeostatic process. The expression patterns of the 4 common differentially expressed miRNAs (miR-202-3p, miR-202-5p, miR-147 and miR-126-3p) were shown in Fig 6e.

### RT-qPCR of candidate miRNAs and genes

**Fig 7:**
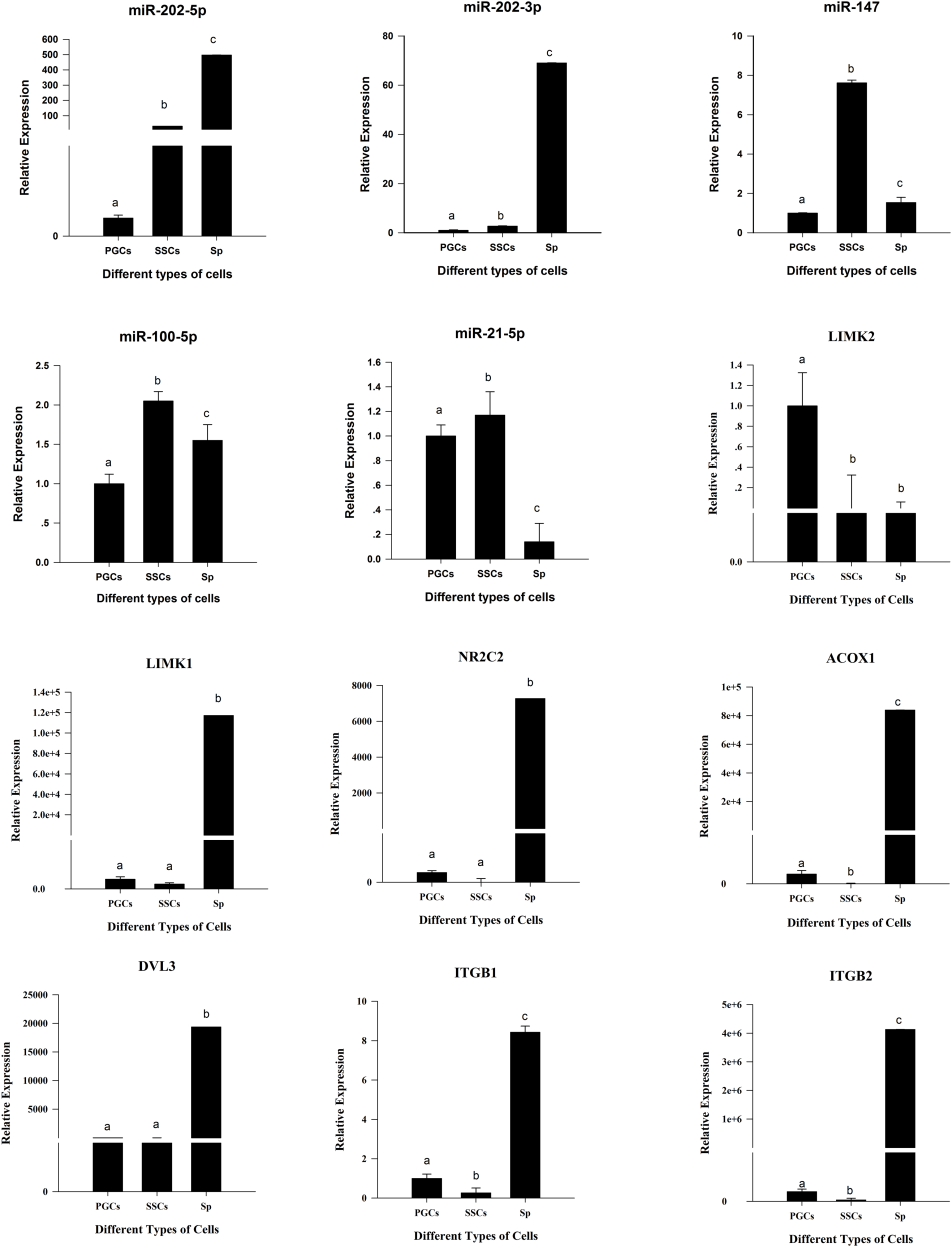
Relative Expression of candidate miRNAs and genes in three types of cells. Lowercase letters indicated *p* <= 0.01.

We assessed the relative expression of the above 7 candidate genes and 5 corresponding miRNAs by RT-qPCR (Fig 7). The SYBR green primers were provided in S4 Table. From this analysis, miR-202-3p/5p showed the highest expression in Sp and miR-147 and miR-100-5p, miR-21-5p present the high expression in SSCs. Furthermore, this trend was in accordance with target genes. We found that 6 genes highly expressed in Sp cells, namely, *LIMK1*, *NR2C2*, *DVL3*, *ACOX1*, *ITGB1* and *ITGB2*. And the expression levels of the former three genes, *LIMK1*, *NR2C2* and *DVL3*, present no significant differences between PGCs and SSCs, while significant differences were found between two groups, PGCs and Sp, and SSCs and Sp. Furthermore, the expression levels of the latter three genes, *ACOX1*, *ITGB1* and *ITGB2*, showed significant differences among three types of cells. In addition, another gene, *LIMK2* expressed higher in PGCs than SSCs and Sp.

### MiR-202-5p bind to *LIMK2* genes in chicken

**Fig 8:**
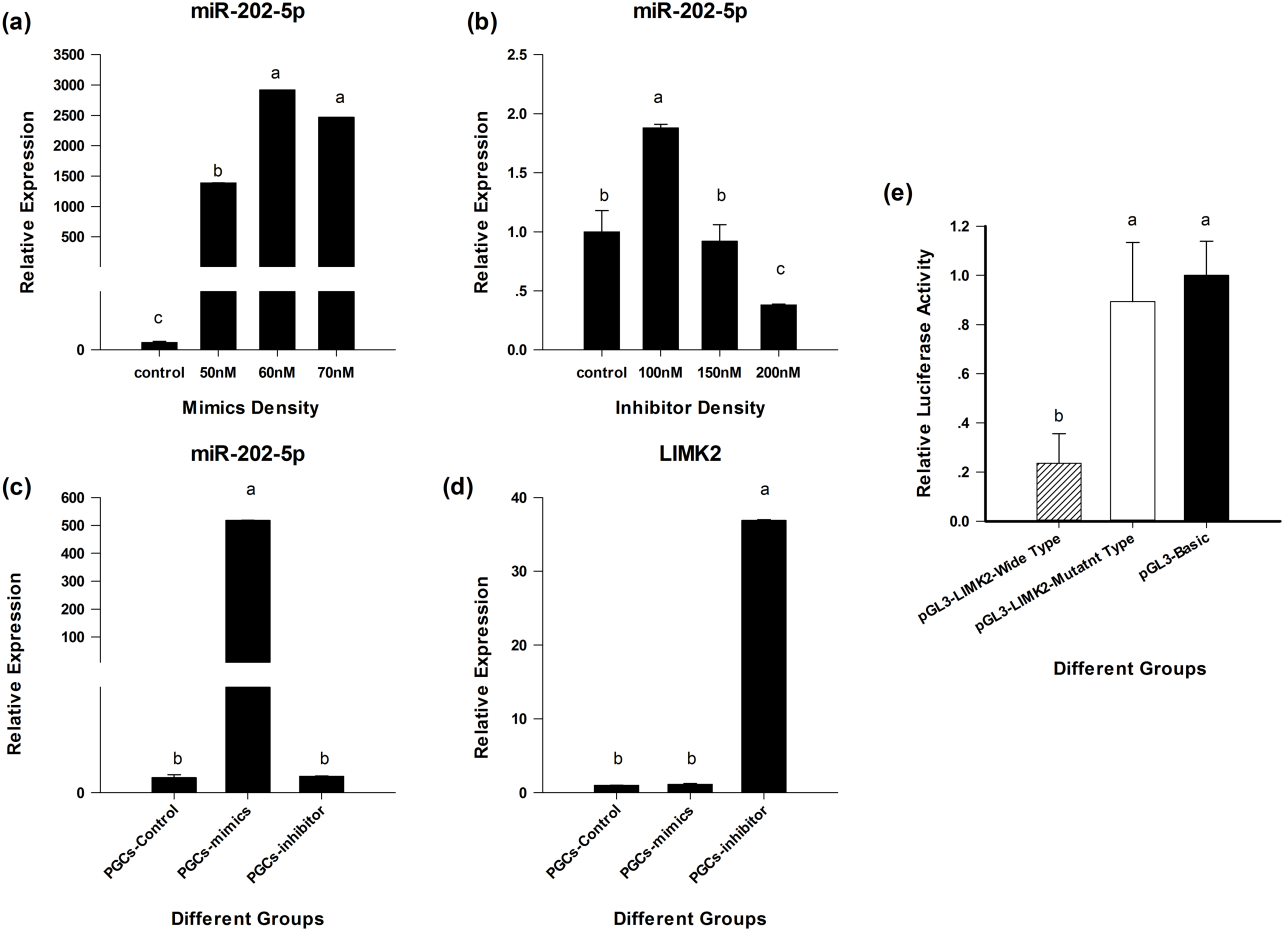
miR-202-5p bind to *LIMK2* genes in chicken. Lowercase letters indicated *p* <= 0.01.

From above the result, we chose miR-202-5p as candidate factor to verify relationship between miR-202-5p and *LIMK2*. In order to verify the relationship between miR-202-5p and *LIMK2*, we transfected the miR-202-5p mimics and inhibitor in DF-1 and PGCs, respectively. First we used DF-1 to investigate the efficiency of miR-202-5p mimic and inhibitor. From this treatment we could ensure these mimic and inhibitor were available. And 60nM for mimics and 200nM for inhibitor were the applicable density for DF-1 (Fig 8a & 8b). Next we performed the same assay to transfected PGCs. The result showed that there was a significant difference between PGCs-mimics and PGCs-inhibitor group and PGCs-control group. In miR-202-5p, PGCs-mimics group was 538.3 times of other two groups but in *LIMK2* the expression was the lowest in three groups (Fig 8c and 8d). And PGCs-inhibitor group was 0.6 times of other two groups, meanwhile, *LIMK2* showed the highest expression in three groups (Fig 8C and 8D). This result indicated that the sequencing result was believable and there was a relationship between miR-202-5p and *LIMK2*.

Also, we performed the Dual-Luciferase assay to detect the relationship between miR-202-5p and *LIMK2*. Compared with control group, the experiment group showed lower fluorescence activity than control group which indicated that miR-202-5p could restrain *LIMK2* transcript regulation (Fig 8e)

## Discussion

In this study, we detected 15 miRNAs that were enriched in the PGCs, SSCs, and Sp libraries and obtained 21 predicted target genes that may play different roles in sperm formation and germ cells development. We also identified several pathways associated with germ line cell development, including Melanogenesis, Steroid hormone biosynthesis. Among them, we identified 12 miRNAs and their target genes that may play important roles in germ line stem cell development and spermiogenesis.

Our Venn analysis of the most abundant mature miRNAs with counts >= 10000 in the PGCs, SSCs, and Sp cells identified 11 miRNAs that were common in the three types of cells, including miR-21-5p and miR-10a-5p. In human, miR-21-5p is as a marker that can be used to evaluate the poor response to *in vitro* fertilization [20]. Further, deep sequencing detected let-7, miR-21, and miR-30 in rainbow trout eggs, which indicated that these miRNAs may play roles in controlling egg quality [21]. Our analysis of the differently expressed miRNAs revealed four miRNA (miR-202-5p/3p, miR-126-3p, miR-147) that were differentially expressed in all three comparisons (SSC vs. PGC, Sp vs. SSC, and Sp vs. PGC). During early testis development, miR-202-5p/3p have been reported to play a conserved expression role in gonad, and *SOX9* was identified as the downstream gene that was the testis-determining factor [22]. In human, miR-202-5p was reported to be up-regulated by 17-fold (P <0.00001) in normal fertile men compared with men with azoospermia [23], which implied that miR-202-5p played an important role in spermatogenesis. Through RT-qPCR and dual-luciferase assay, we confirmed miR-202-5p bind to the *LIMK2* in chicken and *LIMK2* showed the highest expression in PGCs. Thus, miR-202-5p have an effect on *LIMK2* and involved in germ cell development. We also found that the 11 miRNAs that were common in the three types of cells showed a significant relationship with spermatogenesis; in particular, miR-21-5p and miR-100-5p, which were present in high abundance in PGCs and SSCs.

We also identified several target genes, such as those encoding *HOXD4* and *DVL3*, which are related to germline stem cell proliferation or spermatogenesis. This finding were similar to Kruger and Zhang. Overexpression of the homeobox transcription factors *Hoxc8* and *Hoxd4* was reported to cause severe cartilage defects as a result of delayed cartilage maturation [24]. *DVL3* is involved in the Notch signaling and Wnt signaling pathways and is a key element in the latter pathway. *Dvl* interprets signals from various receptors and transmits them to different effector molecules in this pathway [25]. *HOXD4* is associated with many processes, including stem cell differentiation. If the expression of *HOX* genes (including *HOXD4*) is repressed in PGCs, the cells cannot differentiate [26]. Furthermore, several *HOX* genes have been shown to be expressed in the posterior lateral line primordium [27], and it has been suggested that the genes encoding *HOXB6* and other *HOX* genes may be related to chicken male germ cell development and differentiation, and to cell metabolism processes [17]. We used 60nM to perform the cell transfection of mimics because there is no difference between 60nM and 70 nM (Fig 8A).

Many complex factors support spermatogenesis and germ cell development. To further investigate the functions of miRNAs and their target genes more experiments need to be performed like, for example, dual luciferase reporter assays to detect germ cells, and miRNA knockdown to identify target genes expression levels.

In conclusion, we identified several novel miRNAs such as miR-202-5p, and their target genes *LIMK2*, which may be involved in spermatogenesis and germline stem cell development in chicken. These data provide a strong foundation for the study of azoospermia in chicken and may contribute to the search for a solution this problem at the molecular levels. We have no competing interests in this research.

## Supporting information

S1 Table The list of frequently microRNA in three types of cells. The letter marked with red were the common of three types of cells.
(DOC)

S2 Table List of KEGG pathways enriched in the target genes of the 11 common miRNAs with counts >= 10000 in the PGCs, SSCs, and Sp libraries. (DOC)

S3 Table List of KEGG pathways enriched in the target genes of the four common differentially expressed miRNAs in three comparisons (SSCs vs. PGCs, Sp vs. SSCs, and Sp vs. PGCs). (DOC)

S4 Table List of primers of seven candidate genes. (DOC)
