# Supplementary material for: Discovery of microRNAs during early spermatogenesis in chicken
Source: PLoS One. 2017 May 22;12(5):e0177098. doi: 10.1371/journal.pone.0177098 (PMC5439670; doi:10.1371/journal.pone.0177098)
Supplement: S1 Table — The letter marked with red were the common of three types of cells. (DOC) [file pone.0177098.s001.doc]

**Discovery of microRNAs during early spermatogenesis in chicken**

Lu Xu^1^†, Qixin Guo ^1^†, Guobin Chang^1^*, Lingling Qiu ^1^, Xiangping Liu^2^, Yulin Bi^1^, Yu Zhang^1^, Hongzhi Wang^2^, Wei Lu^1^, Lichen Ren^1^, Ying Chen^1^, Yang Zhang^1^, Qi Xu^1^, Guohong Chen^1^*

^1^College of Animal Science and Technology, Yangzhou University, Yangzhou, Jiangsu 225009, China

^2^Poultry Institute, Chinese Academy of Agricultural Sciences, Yangzhou, Jiangsu 225003, China

† These authors contributed equally to this work.

Email addresses: Lu Xu: [herry2800@163.com](mailto:herry2800@163.com); Qixin Guo: [scoot304@163.com](mailto:scoot304@163.com); Lingling Qiu: [260059396@qq.com](mailto:260059396@qq.com); Xiangping Liu: [983036654@qq.com](mailto:983036654@qq.com) Yulin Bi: [410681572@qq.com](mailto:410681572@qq.com); Yu Zhang: [yuzhang@yzu.edu.cn](mailto:yuzhang@yzu.edu.cn); Hongzhi Wang: [434373554@qq.com](mailto:434373554@qq.com); Wei Lu: 759145237@qq.com; Lichen Ren: 515656223@qq.com; Ying Chen: 984662816@qq.com; Yang Zhang: [629911642@qq.com](mailto:629911642@qq.com); Qi Xu: [xuqi@yzu.edu.cn](mailto:xuqi@yzu.edu.cn);

Table The list of frequently microRNA in three types of cells

| Table 2 The list of frequently microRNA in three types of cells | | | | |
| --- | --- | --- | --- | --- |
| Cells | Feature ID | Count | Name | sequence |
| PGCs | mir-21 (Gallus gallus) | 3239094 | miR-21-5p | UAGCUUAUCAGACUGAUGUUGA |
|  | mir-22 (Gallus gallus) | 1564979 | miR-22-3p | AAGCUGCCAGUUGAAGAACUGU |
|  | mir-10a (Gallus gallus) | 1217410 | miR-10a-5p | UACCCUGUAGAUCCGAAUUUGU |
|  | mir-10b (Gallus gallus) | 1192816 | miR-10b-5p | UACCCUGUAGAACCGAAUUUGU |
|  | mir-92-1 (Gallus gallus) | 628276 | miR-92-3p | UAUUGCACUUGUCCCGGCCUG |
|  | mir-26a (Gallus gallus) | 408672 | miR-26a-5p | UUCAAGUAAUCCAGGAUAGGC |
|  | mir-148a (Gallus gallus) | 373824 | miR-148a-3p | UCAGUGCACUACAGAACUUUGU |
|  | mir-181a-2 (Gallus gallus) | 363553 | miR-181a-5p | AACAUUCAACGCUGUCGGUGAGU |
|  | mir-181a-1 (Gallus gallus) | 363542 | miR-181a-5p | AACAUUCAACGCUGUCGGUGAGU |
|  | mir-30d (Gallus gallus) | 243825 | miR-30d | UGUAAACAUCCCCGACUGGAAG |
|  | mir-146c (Gallus gallus) | 205973 | miR-146c-5p | UGAGAACUGAAUUCCAUGGACUG |
|  | mir-454 (Gallus gallus) | 184717 | miR-454-3p | UAGUGCAAUAUUGCUUAUAGGGU |
|  | mir-30a (Gallus gallus) | 156769 | miR-30a-5p | UGUAAACAUCCUCGACUGGAAG |
|  | mir-222a (Gallus gallus) | 148375 | miR-222a | AGCUACAUCUGGCUACUGGGUCUC |
|  | mir-125b-2 (Gallus gallus) | 142280 | miR-125b-5p | UCCCUGAGACCCUAACUUGUGA |
|  | let-7f (Gallus gallus) | 120512 | let-7f-5p | UGAGGUAGUAGAUUGUAUAGUU |
|  | mir-100 (Gallus gallus) | 109098 | miR-100-5p | AACCCGUAGAUCCGAACUUGUG |
|  | mir-27b (Gallus gallus) | 88030 | miR-27b-3p | UUCACAGUGGCUAAGUUCUGC |
|  | mir-181b-2 (Gallus gallus) | 78817 | miR-181b-5p | AACAUUCAUUGCUGUCGGUGGG |
|  | mir-181b-1 (Gallus gallus) | 78802 | miR-181b-5p | AACAUUCAUUGCUGUCGGUGGG |
|  | mir-456 (Gallus gallus) | 71296 | miR-456-3p | CAGGCUGGUUAGAUGGUUGUCA |
|  | mir-130a (Gallus gallus) | 65585 | miR-130a-3p | CAGUGCAAUAUUAAAAGGGCAU |
|  | mir-31 (Gallus gallus) | 61987 | miR-31-5p | AGGCAAGAUGUUGGCAUAGCUG |
|  | mir-2954 (Gallus gallus) | 58563 | miR-2954 | CAUCCCCAUUCCACUCCUAGCA |
|  | mir-140 (Gallus gallus) | 53565 | miR-140-3p | CCACAGGGUAGAACCACGGAC |
|  | mir-199-2 (Gallus gallus) | 49161 | miR-199-3p | UACAGUAGUCUGCACAUUGG |
|  | mir-130b (Gallus gallus) | 49149 | miR-130b-3p | CAGUGCAAUAAUGAAAGGGCGU |
|  | mir-199-1 (Gallus gallus) | 49148 | miR-199-3p | UACAGUAGUCUGCACAUUGG |
|  | mir-30c-2 (Gallus gallus) | 47459 | miR-30c-5p | UGUAAACAUCCUACACUCUCAGCU |
|  | mir-30c-1 (Gallus gallus) | 47420 | miR-30c-5p | UGUAAACAUCCUACACUCUCAGCU |
|  | mir-1559 (Gallus gallus) | 47344 | miR-1559-5p | UUCGAUGCUUGUAUGCUACUCC |
|  | mir-221 (Gallus gallus) | 43741 | miR-221-3p | AGCUACAUUGUCUGCUGGGUUUC |
|  | mir-103-2 (Gallus gallus) | 36389 | miR-103-3p | AGCAGCAUUGUACAGGGCUAUGA |
|  | mir-301a (Gallus gallus) | 36120 | miR-301a-3p | CAGUGCAAUAAUAUUGUCAAAGCAU |
|  | mir-103-1 (Gallus gallus) | 36110 | miR-103-3p | AGCAGCAUUGUACAGGGCUAUGA |
|  | mir-15c (Gallus gallus) | 29537 | miR-15c-5p | UAGCAGCACAUCAUGGUUUGUA |
|  | mir-21 (Gallus gallus) | 28311 | miR-21-3p | CAACAACAGUCGGUAGGCUGUC |
|  | mir-301b (Gallus gallus) | 24239 | miR-301b-3p | CAGUGCAAUAGUAUUGUCAAAGCAU |
|  | mir-130c (Gallus gallus) | 23831 | miR-130c-3p | CAGUGCAAUGUUAAAAGGGCAU |
|  | mir-128-1 (Gallus gallus) | 21667 | miR-128-3p | UCACAGUGAACCGGUCUCUUU |
|  | mir-128-2 (Gallus gallus) | 20980 | miR-128-3p | UCACAGUGAACCGGUCUCUUU |
|  | mir-16-1 (Gallus gallus) | 19681 | miR-16-5p | UAGCAGCACGUAAAUAUUGGUG |
|  | mir-16-2 (Gallus gallus) | 19676 | miR-16-5p | UAGCAGCACGUAAAUAUUGGUG |
|  | mir-19b (Gallus gallus) | 19033 | miR-19b-3p | UGUGCAAAUCCAUGCAAAACUGA |
|  | mir-181a-1 (Gallus gallus) | 18980 | miR-181a-3p | ACCAUCGACCGUUGAUUGUACC |
|  | mir-455 (Gallus gallus) | 18811 | miR-455-5p | UAUGUGCCCUUGGACUACAUCG |
|  | mir-101-1 (Gallus gallus) | 18760 | miR-101-3p | GUACAGUACUGUGAUAACUGAA |
|  | mir-101-2 (Gallus gallus) | 18491 | miR-101-3p | GUACAGUACUGUGAUAACUGAA |
|  | let-7a-2 (Gallus gallus) | 17505 | let-7a-5p | UGAGGUAGUAGGUUGUAUAGUU |
|  | let-7a-3 (Gallus gallus) | 17469 | let-7a-5p | UGAGGUAGUAGGUUGUAUAGUU |
|  | let-7j (Gallus gallus) | 17441 | let-7j-5p | UGAGGUAGUAGGUUGUAUAGUU |
|  | let-7a-1 (Gallus gallus) | 17426 | let-7a-5p | UGAGGUAGUAGGUUGUAUAGUU |
|  | mir-219b (Gallus gallus) | 16639 | miR-219b | CACAAGAAUUGCGUUUGGACAA |
|  | mir-17 (Gallus gallus) | 14407 | miR-17-5p | CAAAGUGCUUACAGUGCAGGUAGU |
|  | let-7i (Gallus gallus) | 14165 | let-7i | UGAGGUAGUAGUUUGUGCUGU |
|  | let-7c (Gallus gallus) | 14080 | let-7c-5p | UGAGGUAGUAGGUUGUAUGGUU |
|  | mir-429 (Gallus gallus) | 13967 | miR-429-3p | UAAUACUGUCUGGUAAUGCCGU |
|  | mir-196-3 (Gallus gallus) | 13760 | miR-196-5p | UAGGUAGUUUCAUGUUGUUGG |
|  | let-7g (Gallus gallus) | 13529 | let-7g-5p | UGAGGUAGUAGUUUGUACAGU |
|  | mir-215 (Gallus gallus) | 12853 | miR-215-5p | AUGACCUAUGAAUUGACAGAC |
|  | mir-107 (Gallus gallus) | 11418 | miR-107-3p | AGCAGCAUUGUACAGGGCUAUCA |
|  | mir-20a (Gallus gallus) | 10975 | miR-20a-5p | UAAAGUGCUUAUAGUGCAGGUAG |
|  | mir-30e (Gallus gallus) | 10820 | miR-30e-3p | UUUCAGUCGGAUGUUUACAGC |
|  | mir-125b-2 (Gallus gallus) | 10771 | miR-125b-3p | ACAAGUCAGGCUCUUGGGACCU |
|  | mir-199-2 (Gallus gallus) | 10676 | miR-199-5p | CCCAGUGUUCAGACUACCUGUUC |
|  | mir-199-1 (Gallus gallus) | 10672 | miR-199-5p | CCCAGUGUUCAGACUACCUGUUC |
|  | mir-205b (Gallus gallus) | 10211 | miR-205b | CCCUUCAUUCCACCGGAAUCUG |
| SSCs | mir-29a (Gallus gallus) | 3681234 | miR-21-5p | UAGCUUAUCAGACUGAUGUUGA |
|  | mir-29a (Gallus gallus) | 2407099 | miR-22-3p | AAGCUGCCAGUUGAAGAACUGU |
|  | mir-29b-1 (Gallus gallus) | 694072 | miR-92-3p | UAUUGCACUUGUCCCGGCCUG |
|  | mir-29b-1 (Gallus gallus) | 582015 | miR-26a-5p | UUCAAGUAAUCCAGGAUAGGC |
|  | let-7i (Gallus gallus) | 532052 | miR-10a-5p | UACCCUGUAGAUCCGAAUUUGU |
|  | mir-135a-2 (Gallus gallus) | 460088 | miR-148a-3p | UCAGUGCACUACAGAACUUUGU |
|  | mir-135a-2 (Gallus gallus) | 360169 | miR-30a-5p | UGUAAACAUCCUCGACUGGAAG |
|  | mir-33-1 (Gallus gallus) | 348514 | miR-30d | UGUAAACAUCCCCGACUGGAAG |
|  | mir-33-1 (Gallus gallus) | 344005 | miR-222a | AGCUACAUCUGGCUACUGGGUCUC |
|  | let-7a-3 (Gallus gallus) | 259172 | miR-100-5p | AACCCGUAGAUCCGAACUUGUG |
|  | let-7a-3 (Gallus gallus) | 229652 | miR-181a-5p | AACAUUCAACGCUGUCGGUGAGU |
|  | let-7b (Gallus gallus) | 229642 | miR-181a-5p | AACAUUCAACGCUGUCGGUGAGU |
|  | mir-99a (Gallus gallus) | 224098 | miR-146c-5p | UGAGAACUGAAUUCCAUGGACUG |
|  | mir-99a (Gallus gallus) | 193723 | miR-125b-5p | UCCCUGAGACCCUAACUUGUGA |
|  | let-7c (Gallus gallus) | 165035 | let-7f-5p | UGAGGUAGUAGAUUGUAUAGUU |
|  | let-7c (Gallus gallus) | 154540 | miR-454-3p | UAGUGCAAUAUUGCUUAUAGGGU |
|  | mir-125b-2 (Gallus gallus) | 103856 | miR-2954 | CAUCCCCAUUCCACUCCUAGCA |
|  | mir-125b-2 (Gallus gallus) | 81409 | let-7k-5p | UGAGGUAGUAGAUUGAAUAGUU |
|  | mir-155 (Gallus gallus) | 81183 | miR-221-3p | AGCUACAUUGUCUGCUGGGUUUC |
|  | mir-222a (Gallus gallus) | 66784 | miR-30c-5p | UGUAAACAUCCUACACUCUCAGCU |
|  | mir-221 (Gallus gallus) | 66712 | miR-30c-5p | UGUAAACAUCCUACACUCUCAGCU |
|  | mir-221 (Gallus gallus) | 64114 | let-7i | UGAGGUAGUAGUUUGUGCUGU |
|  | mir-92-1 (Gallus gallus) | 59477 | miR-27b-3p | UUCACAGUGGCUAAGUUCUGC |
|  | mir-92-1 (Gallus gallus) | 56551 | miR-456-3p | CAGGCUGGUUAGAUGGUUGUCA |
|  | mir-19b (Gallus gallus) | 54886 | miR-199-3p | UACAGUAGUCUGCACAUUGG |
|  | mir-19b (Gallus gallus) | 54861 | miR-199-3p | UACAGUAGUCUGCACAUUGG |
|  | mir-20a (Gallus gallus) | 53568 | miR-1559-5p | UUCGAUGCUUGUAUGCUACUCC |
|  | mir-20a (Gallus gallus) | 53137 | miR-140-3p | CCACAGGGUAGAACCACGGAC |
|  | mir-19a (Gallus gallus) | 51727 | let-7a-5p | UGAGGUAGUAGGUUGUAUAGUU |
|  | mir-19a (Gallus gallus) | 51645 | let-7a-5p | UGAGGUAGUAGGUUGUAUAGUU |
|  | mir-18a (Gallus gallus) | 51569 | let-7j-5p | UGAGGUAGUAGGUUGUAUAGUU |
|  | mir-18a (Gallus gallus) | 51518 | let-7a-5p | UGAGGUAGUAGGUUGUAUAGUU |
|  | mir-17 (Gallus gallus) | 48374 | let-7c-5p | UGAGGUAGUAGGUUGUAUGGUU |
|  | mir-17 (Gallus gallus) | 46993 | miR-130a-3p | CAGUGCAAUAUUAAAAGGGCAU |
|  | mir-16-1 (Gallus gallus) | 41428 | miR-202-5p | UUUCCUAUGCAUAUACUUCUUU |
|  | mir-16-1 (Gallus gallus) | 39174 | miR-130b-3p | CAGUGCAAUAAUGAAAGGGCGU |
|  | mir-15a (Gallus gallus) | 37863 | miR-181b-5p | AACAUUCAUUGCUGUCGGUGGG |
|  | mir-26a (Gallus gallus) | 37850 | miR-181b-5p | AACAUUCAUUGCUGUCGGUGGG |
|  | mir-26a (Gallus gallus) | 37435 | let-7g-5p | UGAGGUAGUAGUUUGUACAGU |
|  | mir-153 (Gallus gallus) | 36602 | miR-15c-5p | UAGCAGCACAUCAUGGUUUGUA |
|  | mir-153 (Gallus gallus) | 33009 | miR-219b | CACAAGAAUUGCGUUUGGACAA |
|  | mir-148a (Gallus gallus) | 31972 | miR-125b-3p | ACAAGUCAGGCUCUUGGGACCU |
|  | mir-148a (Gallus gallus) | 31124 | miR-101-3p | GUACAGUACUGUGAUAACUGAA |
|  | mir-196-2 (Gallus gallus) | 30538 | miR-101-3p | GUACAGUACUGUGAUAACUGAA |
|  | mir-196-2 (Gallus gallus) | 30403 | miR-21-3p | CAACAACAGUCGGUAGGCUGUC |
|  | mir-138-1 (Gallus gallus) | 27596 | miR-103-3p | AGCAGCAUUGUACAGGGCUAUGA |
|  | mir-138-1 (Gallus gallus) | 27430 | miR-103-3p | AGCAGCAUUGUACAGGGCUAUGA |
|  | mir-128-2 (Gallus gallus) | 25973 | miR-128-3p | UCACAGUGAACCGGUCUCUUU |
|  | mir-128-2 (Gallus gallus) | 25826 | let-7b | UGAGGUAGUAGGUUGUGUGGUU |
|  | mir-187 (Gallus gallus) | 24412 | miR-128-3p | UCACAGUGAACCGGUCUCUUU |
|  | mir-187 (Gallus gallus) | 23074 | miR-130c-3p | CAGUGCAAUGUUAAAAGGGCAU |
|  | mir-32 (Gallus gallus) | 23058 | miR-146b-5p | UGAGAACUGAAUUCCAUAGGCG |
|  | mir-32 (Gallus gallus) | 21866 | miR-19b-3p | UGUGCAAAUCCAUGCAAAACUGA |
|  | mir-133a-1 (Gallus gallus) | 20889 | miR-147 | GUGUGCGGAAAUGCUUCUGC |
|  | mir-133a-1 (Gallus gallus) | 19878 | miR-301a-3p | CAGUGCAAUAAUAUUGUCAAAGCAU |
|  | mir-1a-2 (Gallus gallus) | 16598 | miR-301b-3p | CAGUGCAAUAGUAUUGUCAAAGCAU |
|  | mir-1a-2 (Gallus gallus) | 16047 | miR-16-5p | UAGCAGCACGUAAAUAUUGGUG |
|  | mir-124a (Gallus gallus) | 16045 | miR-16-5p | UAGCAGCACGUAAAUAUUGGUG |
|  | mir-124a (Gallus gallus) | 14406 | miR-99a-5p | AACCCGUAGAUCCGAUCUUGUG |
|  | mir-30d (Gallus gallus) | 13317 | miR-29a-3p | UAGCACCAUUUGAAAUCGGUU |
|  | mir-30b (Gallus gallus) | 13177 | miR-17-5p | CAAAGUGCUUACAGUGCAGGUAGU |
|  | mir-30b (Gallus gallus) | 13098 | miR-30b-5p | UGUAAACAUCCUACACUCAGCU |
|  | mir-216a (Gallus gallus) | 12957 | miR-30e-3p | UUUCAGUCGGAUGUUUACAGC |
|  | mir-217 (Gallus gallus) | 12671 | miR-455-5p | UAUGUGCCCUUGGACUACAUCG |
|  | mir-217 (Gallus gallus) | 10960 | miR-31-5p | AGGCAAGAUGUUGGCAUAGCUG |
|  | mir-194 (Gallus gallus) | 10804 | miR-181a-3p | ACCAUCGACCGUUGAUUGUACC |
|  | mir-215 (Gallus gallus) | 10235 | miR-107-3p | AGCAGCAUUGUACAGGGCUAUCA |
| Sp | mir-100 (Gallus gallus) | 61342 | miR-100-5p | AACCCGUAGAUCCGAACUUGUG |
|  | mir-202 (Gallus gallus) | 60108 | miR-202-5p | UUUCCUAUGCAUAUACUUCUUU |
|  | mir-22 (Gallus gallus) | 50181 | miR-22-3p | AAGCUGCCAGUUGAAGAACUGU |
|  | mir-10a (Gallus gallus) | 36530 | miR-10a-5p | UACCCUGUAGAUCCGAAUUUGU |
|  | mir-30d (Gallus gallus) | 33206 | miR-30d | UGUAAACAUCCCCGACUGGAAG |
|  | mir-148a (Gallus gallus) | 25023 | miR-148a-3p | UCAGUGCACUACAGAACUUUGU |
|  | mir-2954 (Gallus gallus) | 21732 | miR-2954 | CAUCCCCAUUCCACUCCUAGCA |
|  | mir-26a (Gallus gallus) | 19018 | miR-26a-5p | UUCAAGUAAUCCAGGAUAGGC |
|  | mir-125b-2 (Gallus gallus) | 18380 | miR-125b-5p | UCCCUGAGACCCUAACUUGUGA |
|  | mir-21 (Gallus gallus) | 18288 | miR-21-5p | UAGCUUAUCAGACUGAUGUUGA |
|  | mir-92-1 (Gallus gallus) | 14421 | miR-92-3p | UAUUGCACUUGUCCCGGCCUG |
|  | let-7f (Gallus gallus) | 11143 | let-7f-5p | UGAGGUAGUAGAUUGUAUAGUU |
