# Supplementary material for: Discovery of microRNAs during early spermatogenesis in chicken
Source: PLoS One. 2017 May 22;12(5):e0177098. doi: 10.1371/journal.pone.0177098 (PMC5439670; doi:10.1371/journal.pone.0177098)
Supplement: S2 Table — (DOC) [file pone.0177098.s002.doc]

**Discovery of microRNAs during early spermatogenesis in chicken**

Lu Xu^1^†, Qixin Guo ^1^†, Guobin Chang^1^*, Lingling Qiu ^1^, Xiangping Liu^2^, Yulin Bi^1^, Yu Zhang^1^, Hongzhi Wang^2^, Wei Lu^1^, Lichen Ren^1^, Ying Chen^1^, Yang Zhang^1^, Qi Xu^1^, Guohong Chen^1^*

^1^College of Animal Science and Technology, Yangzhou University, Yangzhou, Jiangsu 225009, China

^2^Poultry Institute, Chinese Academy of Agricultural Sciences, Yangzhou, Jiangsu 225003, China

† These authors contributed equally to this work.

Email addresses: Lu Xu: [herry2800@163.com](mailto:herry2800@163.com); Qixin Guo: [scoot304@163.com](mailto:scoot304@163.com); Lingling Qiu: [260059396@qq.com](mailto:260059396@qq.com); Xiangping Liu: [983036654@qq.com](mailto:983036654@qq.com) Yulin Bi: [410681572@qq.com](mailto:410681572@qq.com); Yu Zhang: [yuzhang@yzu.edu.cn](mailto:yuzhang@yzu.edu.cn); Hongzhi Wang: [434373554@qq.com](mailto:434373554@qq.com); Wei Lu: 759145237@qq.com; Lichen Ren: 515656223@qq.com; Ying Chen: 984662816@qq.com; Yang Zhang: [629911642@qq.com](mailto:629911642@qq.com); Qi Xu: [xuqi@yzu.edu.cn](mailto:xuqi@yzu.edu.cn);

Table The list of pathways in three types of cells

| No. | Pathway Name | Hits | Total | Percent | Enrichment Test P_value |
| --- | --- | --- | --- | --- | --- |
| 1 | Adherens junction | 5 | 63 | 7.94% | 0.0057 |
| 2 | Adipocytokine signaling pathway | 2 | 53 | 3.77% | 0.2364 |
| 3 | Alanine. aspartate and glutamate metabolism | 3 | 25 | 12.00% | 0.0113 |
| 4 | alpha-Linolenic acid metabolism | 1 | 15 | 6.67% | 0.2378 |
| 5 | Aminoacyl-tRNA biosynthesis | 2 | 36 | 5.56% | 0.1338 |
| 6 | Apoptosis | 1 | 68 | 1.47% | 0.6904 |
| 7 | Arginine and proline metabolism | 3 | 35 | 8.57% | 0.0258 |
| 8 | Biosynthesis of unsaturated fatty acids | 3 | 18 | 16.67% | 0.005 |
| 9 | Biotin metabolism | 1 | 3 | 33.33% | 0.0656 |
| 10 | Butanoate metabolism | 2 | 25 | 8.00% | 0.0752 |
| 11 | Caffeine metabolism | 1 | 5 | 20.00% | 0.0968 |
| 12 | Calcium signaling pathway | 7 | 139 | 5.04% | 0.0119 |
| 13 | Cardiac muscle contraction | 2 | 49 | 4.08% | 0.2116 |
| 14 | Cell adhesion molecules (CAMs) | 4 | 93 | 4.30% | 0.0811 |
| 15 | Cell cycle | 3 | 103 | 2.91% | 0.2642 |
| 16 | Citrate cycle (TCA cycle) | 1 | 26 | 3.85% | 0.3677 |
| 17 | Cysteine and methionine metabolism | 1 | 30 | 3.33% | 0.4092 |
| 18 | Cytokine-cytokine receptor interaction | 1 | 163 | 0.61% | 0.9387 |
| 19 | Drug metabolism other enzymes | 1 | 28 | 3.57% | 0.3888 |
| 20 | ECM-receptor interaction | 7 | 69 | 10.14% | 3.00E-04 |
| 21 | Endocytosis | 2 | 172 | 1.16% | 0.7934 |
| 22 | Ether lipid metabolism | 1 | 25 | 4.00% | 0.3568 |
| 23 | Fatty acid biosynthesis | 1 | 5 | 20.00% | 0.0968 |
| 24 | Fatty acid metabolism | 1 | 27 | 3.70% | 0.3783 |
| 25 | Focal adhesion | 8 | 166 | 4.82% | 0.0094 |
| 26 | Galactose metabolism | 1 | 25 | 4.00% | 0.3568 |
| 27 | Gap junction | 3 | 74 | 4.05% | 0.1401 |
| 28 | Glycerolipid metabolism | 1 | 44 | 2.27% | 0.5343 |
| 29 | Glycosaminoglycan biosynthesis chondroitin sulfate | 2 | 20 | 10.00% | 0.0522 |
| 30 | Glycosphingolipid biosynthesis ganglio series | 2 | 18 | 11.11% | 0.0439 |
| 31 | Glycosphingolipid biosynthesis lacto and neolacto series | 1 | 18 | 5.56% | 0.2756 |
| 32 | Glycosylphosphatidylinositol(GPI)-anchor biosynthesis | 2 | 18 | 11.11% | 0.0439 |
| 33 | GnRH signaling pathway | 3 | 75 | 4.00% | 0.144 |
| 34 | Hedgehog signaling pathway | 1 | 42 | 2.38% | 0.5182 |
| 35 | Heparan sulfate biosynthesis | 1 | 22 | 4.55% | 0.3232 |
| 36 | Histidine metabolism | 1 | 18 | 5.56% | 0.2756 |
| 37 | Homologous recombination | 1 | 22 | 4.55% | 0.3232 |
| 38 | Inositol phosphate metabolism | 2 | 44 | 4.55% | 0.181 |
| 39 | Insulin signaling pathway | 4 | 106 | 3.77% | 0.1148 |
| 40 | Intestinal immune network for IgA production | 1 | 29 | 3.45% | 0.3991 |
| 41 | Jak-STAT signaling pathway | 3 | 111 | 2.70% | 0.3008 |
| 42 | Limonene and pinene degradation | 1 | 6 | 16.67% | 0.112 |
| 43 | Lysine degradation | 1 | 33 | 3.03% | 0.4385 |
| 44 | Lysosome | 3 | 91 | 3.30% | 0.2106 |
| 45 | MAPK signaling pathway | 3 | 204 | 1.47% | 0.6795 |
| 46 | Melanogenesis | 6 | 83 | 7.23% | 0.0039 |
| 47 | Metabolic pathways | 30 | 842 | 3.56% | 2.00E-04 |
| 48 | mTOR signaling pathway | 1 | 41 | 2.44% | 0.5099 |
| 49 | N-Glycan biosynthesis | 2 | 37 | 5.41% | 0.1396 |
| 50 | Natural killer cell mediated cytotoxicity | 1 | 66 | 1.52% | 0.6797 |
| 51 | Neuroactive ligand-receptor interaction | 4 | 264 | 1.52% | 0.6628 |
| 52 | Nicotinate and nicotinamide metabolism | 1 | 18 | 5.56% | 0.2756 |
| 53 | Notch signaling pathway | 2 | 42 | 4.76% | 0.169 |
| 54 | Nucleotide excision repair | 1 | 31 | 3.23% | 0.4191 |
| 55 | O-Glycan biosynthesis | 2 | 26 | 7.69% | 0.0801 |
| 56 | Oocyte meiosis | 2 | 89 | 2.25% | 0.4543 |
| 57 | Oxidative phosphorylation | 1 | 108 | 0.93% | 0.8434 |
| 58 | Peroxisome | 3 | 64 | 4.69% | 0.1033 |
| 59 | Phenylalanine metabolism | 1 | 13 | 7.69% | 0.2115 |
| 60 | Phosphatidylinositol signaling system | 2 | 61 | 3.28% | 0.2864 |
| 61 | Porphyrin and chlorophyll metabolism | 1 | 19 | 5.26% | 0.2878 |
| 62 | PPAR signaling pathway | 3 | 53 | 5.66% | 0.0681 |
| 63 | Primary bile acid biosynthesis | 1 | 13 | 7.69% | 0.2115 |
| 64 | Progesterone-mediated oocyte maturation | 1 | 69 | 1.45% | 0.6956 |
| 65 | Purine metabolism | 3 | 118 | 2.54% | 0.333 |
| 66 | Pyrimidine metabolism | 1 | 76 | 1.32% | 0.7298 |
| 67 | Regulation of actin cytoskeleton | 5 | 167 | 2.99% | 0.1649 |
| 68 | Retinol metabolism | 1 | 26 | 3.85% | 0.3677 |
| 69 | RNA degradation | 1 | 45 | 2.22% | 0.5421 |
| 70 | RNA polymerase | 1 | 21 | 4.76% | 0.3116 |
| 71 | Sphingolipid metabolism | 1 | 34 | 2.94% | 0.448 |
| 72 | Spliceosome | 2 | 97 | 2.06% | 0.4982 |
| 73 | Starch and sucrose metabolism | 1 | 25 | 4.00% | 0.3568 |
| 74 | Steroid biosynthesis | 1 | 12 | 8.33% | 0.198 |
| 75 | Steroid hormone biosynthesis | 3 | 26 | 11.54% | 0.0125 |
| 76 | Synthesis and degradation of ketone bodies | 2 | 7 | 28.57% | 0.0094 |
| 77 | Terpenoid biosynthesis | 1 | 12 | 8.33% | 0.198 |
| 78 | TGF-beta signaling pathway | 3 | 68 | 4.41% | 0.1175 |
| 79 | Tight junction | 2 | 107 | 1.87% | 0.5497 |
| 80 | Toll-like receptor signaling pathway | 3 | 75 | 4.00% | 0.144 |
| 81 | Tyrosine metabolism | 2 | 28 | 7.14% | 0.0902 |
| 82 | Ubiquitin mediated proteolysis | 3 | 113 | 2.65% | 0.31 |
| 83 | Vascular smooth muscle contraction | 3 | 94 | 3.19% | 0.2238 |
| 84 | VEGF signaling pathway | 1 | 58 | 1.72% | 0.6329 |
| 85 | Wnt signaling pathway | 5 | 122 | 4.10% | 0.0636 |
| 86 | \Glycine. serine and threonine metabolism\ | 1 | 25 | 4.00% | 0.3568 |
| 87 | \Phenylalanine. tyrosine and tryptophan biosynthesis\ | 1 | 5 | 20.00% | 0.0968 |
| 88 | \Valine. leucine and isoleucine degradation\ | 2 | 36 | 5.56% | 0.1338 |
