# Supplementary material for: Discovery of microRNAs during early spermatogenesis in chicken
Source: PLoS One. 2017 May 22;12(5):e0177098. doi: 10.1371/journal.pone.0177098 (PMC5439670; doi:10.1371/journal.pone.0177098)
Supplement: S3 Table — (DOC) [file pone.0177098.s003.doc]

**Discovery of microRNAs during early spermatogenesis in chicken**

Lu Xu^1^†, Qixin Guo ^1^†, Guobin Chang^1^*, Lingling Qiu ^1^, Xiangping Liu^2^, Yulin Bi^1^, Yu Zhang^1^, Hongzhi Wang^2^, Wei Lu^1^, Lichen Ren^1^, Ying Chen^1^, Yang Zhang^1^, Qi Xu^1^, Guohong Chen^1^*

^1^College of Animal Science and Technology, Yangzhou University, Yangzhou, Jiangsu 225009, China

^2^Poultry Institute, Chinese Academy of Agricultural Sciences, Yangzhou, Jiangsu 225003, China

† These authors contributed equally to this work.

Email addresses: Lu Xu: [herry2800@163.com](mailto:herry2800@163.com); Qixin Guo: [scoot304@163.com](mailto:scoot304@163.com); Lingling Qiu: [260059396@qq.com](mailto:260059396@qq.com); Xiangping Liu: [983036654@qq.com](mailto:983036654@qq.com) Yulin Bi: [410681572@qq.com](mailto:410681572@qq.com); Yu Zhang: [yuzhang@yzu.edu.cn](mailto:yuzhang@yzu.edu.cn); Hongzhi Wang: [434373554@qq.com](mailto:434373554@qq.com); Wei Lu: 759145237@qq.com; Lichen Ren: 515656223@qq.com; Ying Chen: 984662816@qq.com; Yang Zhang: [629911642@qq.com](mailto:629911642@qq.com); Qi Xu: [xuqi@yzu.edu.cn](mailto:xuqi@yzu.edu.cn);

Table The list of pathways in three groups

| No. | Pathway Name | Hits | Total | Percent | Q_value |
| --- | --- | --- | --- | --- | --- |
| 1 | ABC transporters | 1 | 31 | 3.23% | 0.0132 |
| 2 | Aminoacyl-tRNA biosynthesis | 2 | 36 | 5.56% | 0.006 |
| 3 | Arachidonic acid metabolism | 1 | 29 | 3.45% | 0.0132 |
| 4 | Calcium signaling pathway - Gallus gallus (chicken) | 2 | 139 | 1.44% | 0.0132 |
| 5 | Cardiac muscle contraction - Gallus gallus (chicken) | 1 | 49 | 2.04% | 0.0149 |
| 6 | Cell adhesion molecules (CAMs) - Gallus gallus (chicken) | 1 | 93 | 1.08% | 0.0174 |
| 7 | Cell cycle | 1 | 103 | 0.97% | 0.0177 |
| 8 | ECM-receptor interaction - Gallus gallus (chicken) | 1 | 69 | 1.45% | 0.0161 |
| 9 | Focal adhesion - Gallus gallus (chicken) | 2 | 166 | 1.20% | 0.0142 |
| 10 | Gap junction - Gallus gallus (chicken) | 1 | 74 | 1.35% | 0.0165 |
| 11 | Glycerolipid metabolism | 1 | 44 | 2.27% | 0.0142 |
| 12 | Glycerophospholipid metabolism | 2 | 61 | 3.28% | 0.0117 |
| 13 | Glyoxylate and dicarboxylate metabolism | 1 | 13 | 7.69% | 0.0126 |
| 14 | Heparan sulfate biosynthesis | 1 | 22 | 4.55% | 0.0126 |
| 15 | Histidine metabolism | 1 | 18 | 5.56% | 0.0126 |
| 16 | Lysine degradation | 1 | 33 | 3.03% | 0.0132 |
| 17 | Lysosome - Gallus gallus (chicken) | 2 | 91 | 2.20% | 0.0126 |
| 18 | MAPK signaling pathway - Gallus gallus (chicken) | 2 | 204 | 0.98% | 0.0158 |
| 19 | Melanogenesis - Gallus gallus (chicken) | 1 | 83 | 1.20% | 0.0174 |
| 20 | Metabolic pathways | 9 | 842 | 1.07% | 0.006 |
| 21 | Neuroactive ligand-receptor interaction - Gallus gallus (chicken) | 1 | 264 | 0.38% | 0.0309 |
| 22 | Notch signaling pathway - Gallus gallus (chicken) | 1 | 42 | 2.38% | 0.0142 |
| 23 | One carbon pool by folate | 1 | 15 | 6.67% | 0.0126 |
| 24 | Oocyte meiosis - Gallus gallus (chicken) | 1 | 89 | 1.12% | 0.0174 |
| 25 | Oxidative phosphorylation | 1 | 108 | 0.93% | 0.0178 |
| 26 | Phosphatidylinositol signaling system - Gallus gallus (chicken) | 1 | 61 | 1.64% | 0.0161 |
| 27 | Porphyrin and chlorophyll metabolism | 1 | 19 | 5.26% | 0.0126 |
| 28 | Progesterone-mediated oocyte maturation - Gallus gallus (chicken) | 1 | 69 | 1.45% | 0.0161 |
| 29 | Protein export | 1 | 20 | 5.00% | 0.0126 |
| 30 | Regulation of actin cytoskeleton - Gallus gallus (chicken) | 4 | 167 | 2.40% | 0.006 |
| 31 | Ribosome | 1 | 69 | 1.45% | 0.0161 |
| 32 | Sphingolipid metabolism | 1 | 34 | 2.94% | 0.0132 |
| 33 | Spliceosome - Gallus gallus (chicken) | 1 | 97 | 1.03% | 0.0174 |
| 34 | Ubiquitin mediated proteolysis | 2 | 113 | 1.77% | 0.0126 |
| 35 | Vascular smooth muscle contraction - Gallus gallus (chicken) | 1 | 94 | 1.06% | 0.0174 |
| 36 | Wnt signaling pathway - Gallus gallus (chicken) | 1 | 122 | 0.82% | 0.019 |
