# Supplementary material for: Discovery of microRNAs during early spermatogenesis in chicken
Source: PLoS One. 2017 May 22;12(5):e0177098. doi: 10.1371/journal.pone.0177098 (PMC5439670; doi:10.1371/journal.pone.0177098)
Supplement: S4 Table — (DOC) [file pone.0177098.s004.doc]

**Discovery of microRNAs during early spermatogenesis in chicken**

Lu Xu^1^†, Qixin Guo ^1^†, Guobin Chang^1^*, Lingling Qiu ^1^, Xiangping Liu^2^, Yulin Bi^1^, Yu Zhang^1^, Hongzhi Wang^2^, Wei Lu^1^, Lichen Ren^1^, Ying Chen^1^, Yang Zhang^1^, Qi Xu^1^, Guohong Chen^1^*

^1^College of Animal Science and Technology, Yangzhou University, Yangzhou, Jiangsu 225009, China

^2^Poultry Institute, Chinese Academy of Agricultural Sciences, Yangzhou, Jiangsu 225003, China

† These authors contributed equally to this work.

Email addresses: Lu Xu: [herry2800@163.com](mailto:herry2800@163.com); Qixin Guo: [scoot304@163.com](mailto:scoot304@163.com); Lingling Qiu: [260059396@qq.com](mailto:260059396@qq.com); Xiangping Liu: [983036654@qq.com](mailto:983036654@qq.com) Yulin Bi: [410681572@qq.com](mailto:410681572@qq.com); Yu Zhang: [yuzhang@yzu.edu.cn](mailto:yuzhang@yzu.edu.cn); Hongzhi Wang: [434373554@qq.com](mailto:434373554@qq.com); Wei Lu: 759145237@qq.com; Lichen Ren: 515656223@qq.com; Ying Chen: 984662816@qq.com; Yang Zhang: [629911642@qq.com](mailto:629911642@qq.com); Qi Xu: [xuqi@yzu.edu.cn](mailto:xuqi@yzu.edu.cn);

Table List of Primers of Seven Candidate Genes

| No. | Gene Name | Primer |
| --- | --- | --- |
| 1 | LIKM1 | Forward: CATGGGAGAGGAGGAAGGGAC  Reverse: AACGCGCCCAGTAGTCCT |
| 2 | LIMK2 | Forward: CTGACTTCGGTCTGTCTCGG  Reverse: GCTTGGCCTCCAAAGCTTAAC |
| 3 | DVL3 | Forward: AATACTGGGGGGAGTCGGGAAAAC  Reverse: TTCACGGTGTGTCGGATGTAGC |
| 4 | NR2C2 | Forward: TCCCGATCATCCTGGTCTGA  Reverse: TCAGTGAACCTGGCAACGTG |
| 5 | ACOX1 | Forward: CGGCGAAAGGAGATCGAGG  Reverse: CCGTCCACGATGAACAAAGC |
| 6 | ITGB1 | Forward: GTGGCCCTGACATCATACCC  Reverse: CAGTTGTCACTGCACTCTTGT |
| 7 | ITGB2 | Forward: TATGGCACCTTCTGCGACTG  Reverse: CACGCAGGCTGCATTCATTT |
